# Chylous ascites and lymphangiectasia in focal segmental glomerulosclerosis – a rare coexistence: a case report

**DOI:** 10.1186/s13256-014-0507-2

**Published:** 2015-02-09

**Authors:** Durjoy Lahiri, Rakesh Agarwal, Manoj Kumar Roy, Amrita Biswas

**Affiliations:** Department of General Medicine, IPGMER and SSKM Hospital, Kolkata, 700020 India

**Keywords:** Chylous ascites, Focal segmental glomerulosclerosis, Lymphangiectasia

## Abstract

**Introduction:**

Nephrotic syndrome is considered a rare cause of chylous ascites. Intestinal lymphangiectasia in a background of chylous ascites and without any lymphatic obstruction has been reported in association with yellow nail syndrome, which is a rare clinical occurrence in itself. The existence of chylous ascites, duodenal and splenic lymphangiectasia (without any lymphatic obstruction) and nephrotic syndrome in the form of focal segmental glomerulosclerosis in the same patient makes this case the first of its kind to be reported in the literature.

**Case presentation:**

Here we report the case of a 54-year-old Asian man who presented with recurrent episodes of anasarca for approximately 25 years. He was subsequently found to have chylous ascites, lymphangiectasia and persistent proteinuria. A renal biopsy revealed focal segmental glomerulosclerosis, not otherwise specified. A lymphangiogram, which was performed with the purpose of addressing the intestinal lymphangiectasia, failed to demonstrate any abnormality of lymphatic channels. He was put on oral steroids with consequent remission of his oedema and proteinuria.

**Conclusions:**

This case highlights the fact that duodenal and splenic lymphangiectasia can exist in a scenario of chylous ascites without any obvious obstruction of lymphatic channels and in the absence of yellow nail syndrome. This case also signifies that chylous ascites may be a rare presenting feature of nephrotic syndrome and hence this aspect should be considered while in diagnostic dilemma regarding such a clinical presentation.

## Introduction

Chylous ascites is defined as the extravasation of milky fluid into the peritoneal cavity. This fluid has high triglyceride (TG) content as the result of leakage of thoracic or intestinal lymph. Chylous ascites is a rare clinical situation; the majority of cases are attributed to obstruction of lymphatic channels due to some cause. Nephrotic syndrome is a rarely reported cause of chylous ascites. The alteration in permeability of serosal and mucosal lymphatics, resulting from the metabolic changes of nephrotic syndrome, is the cause of chylous ascites in such a scenario. The coexistence of duodenal and splenic lymphangiectasia and chylous ascites in a patient with nephrotic syndrome is a rare occurrence. We describe the case of a 54-year-old man presenting with chylous ascites, lymphangiectasia and nephrotic syndrome with focal segmental glomerulosclerotic lesion in his kidney. This kind of association is probably the first of its kind to be reported.

## Case presentation

A 54-year-old non-diabetic and non-hypertensive Asian man presented to us with the complaint of recurrent episodes of bilateral pedal pitting swelling along with abdominal distension and periorbital oedema for the preceding 25 years approximately. The swelling subsides when he visits a local physician and receives a short course of diuretics. There was no associated history of dyspnoea, jaundice, upper gastrointestinal (GI) bleed or recurrent episodes of diarrhoea.

**Figure 1:**
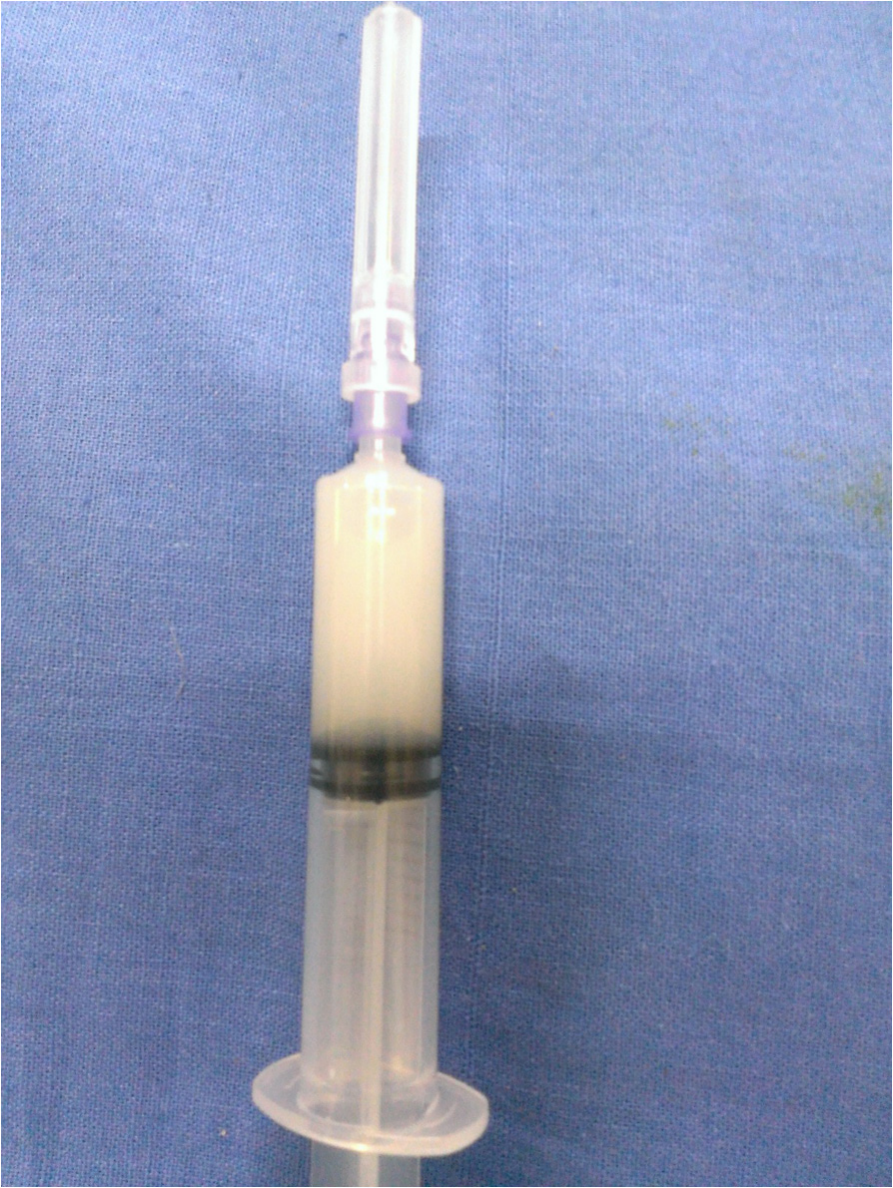
**Milky white colour of the aspirated ascitic fluid.**

**Figure 2:**
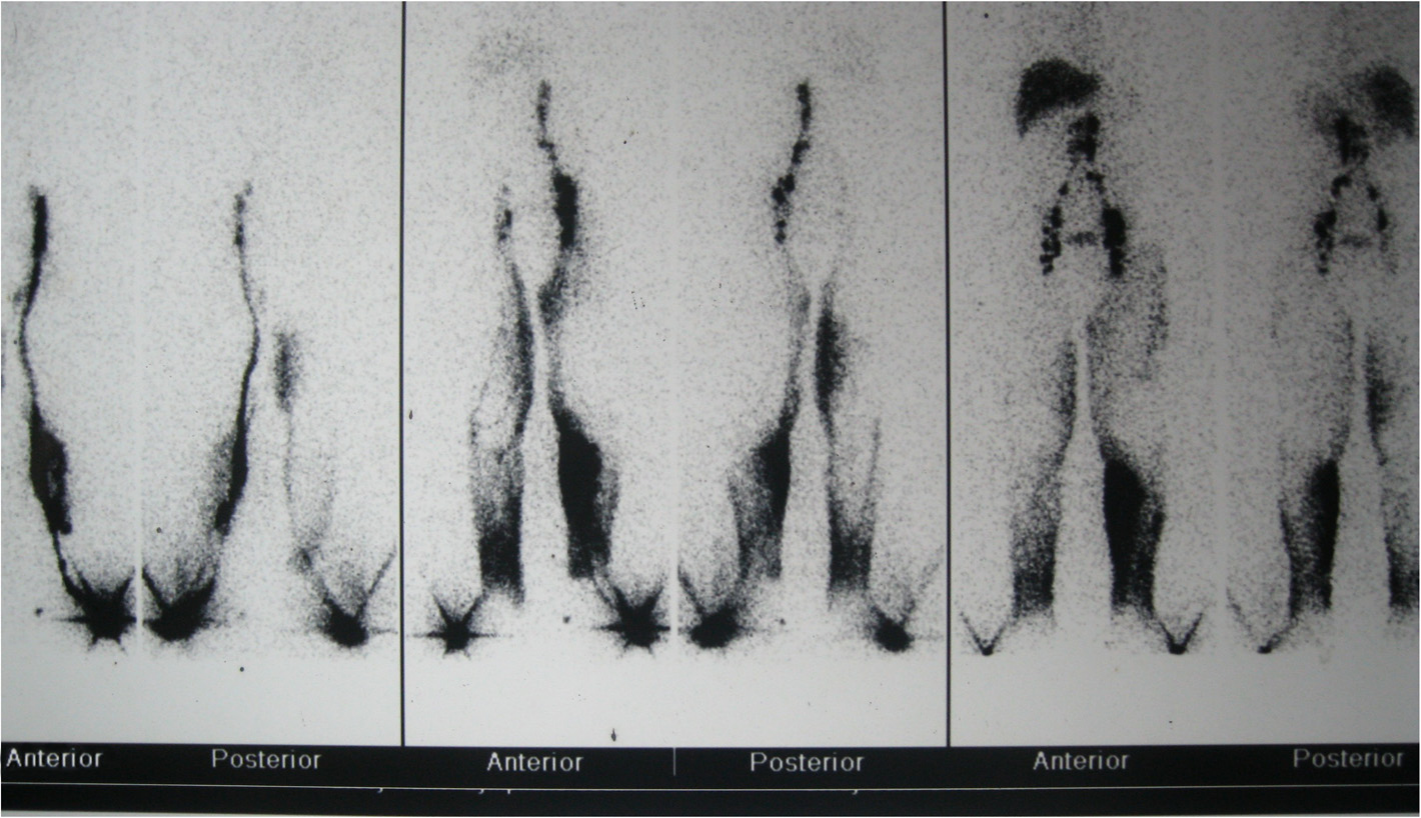
**Lymphangiogram shows no evidence of any obvious lymphatic obstruction.**

**Figure 3:**
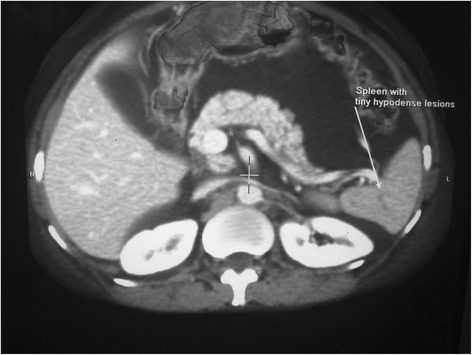
**Contrast-enhanced computed tomography scan of abdomen shows splenic lymphangiectasia.**

A physical examination revealed bilateral pedal oedema, periorbital puffiness, ascites (grade 2), bilateral pleural effusion and mild pallor. He was investigated thoroughly to delineate the cause of anasarca. His blood reports were as follows: haemoglobin 10gm%, serum albumin 1.5gm/dL and serum globulin 3.0gm/dL. Renal function tests revealed urea 30mg% and creatinine 0.9mg/dL. Electrolytes, bilirubin and liver enzymes were within normal limits. His coagulation profile is as follows: prothrombin time 12 seconds (control 12 seconds) and activated partial thromboplastin time 36 seconds (control 35 seconds). Fasting lipid profile showed hypertriglyceridaemia and hypercholesterolaemia. Urine routine examination was 3+ for albumin; his 24-hour urine for albumin was 660mg, with urine output of 800mL in 24 hours. His previous medical records showed higher magnitude of proteinuria and on a few occasions it was approximately 1500mg in 24 hours. An abdominal diagnostic paracentesis was done which strikingly showed milky white colour (Figure 1). Routine analysis of ascitic fluid revealed: cell count 154/mm^3^ (lymphocyte predominant); serum–ascitic albumin gradient 1.2; and adenosine deaminase 6u/L and TG 213mg%. Ascitic fluid culture for bacteria including *Mycobacterium tuberculosis* was negative. A lymphangiogram was done to find the presence of any obstruction in his lymphatic system, which turned out to be normal (Figure 2). A contrast-enhanced computed tomography scan of his abdomen revealed no neoplasm but did reveal the existence of splenic lymphangiectasia (Figure 3). An upper GI endoscopy was performed to rule out any feature of portal hypertension; it revealed duodenal lymphangiectasia. His echocardiography was within normal limits, ruling out cardiac cause of ascites.

**Figure 4:**
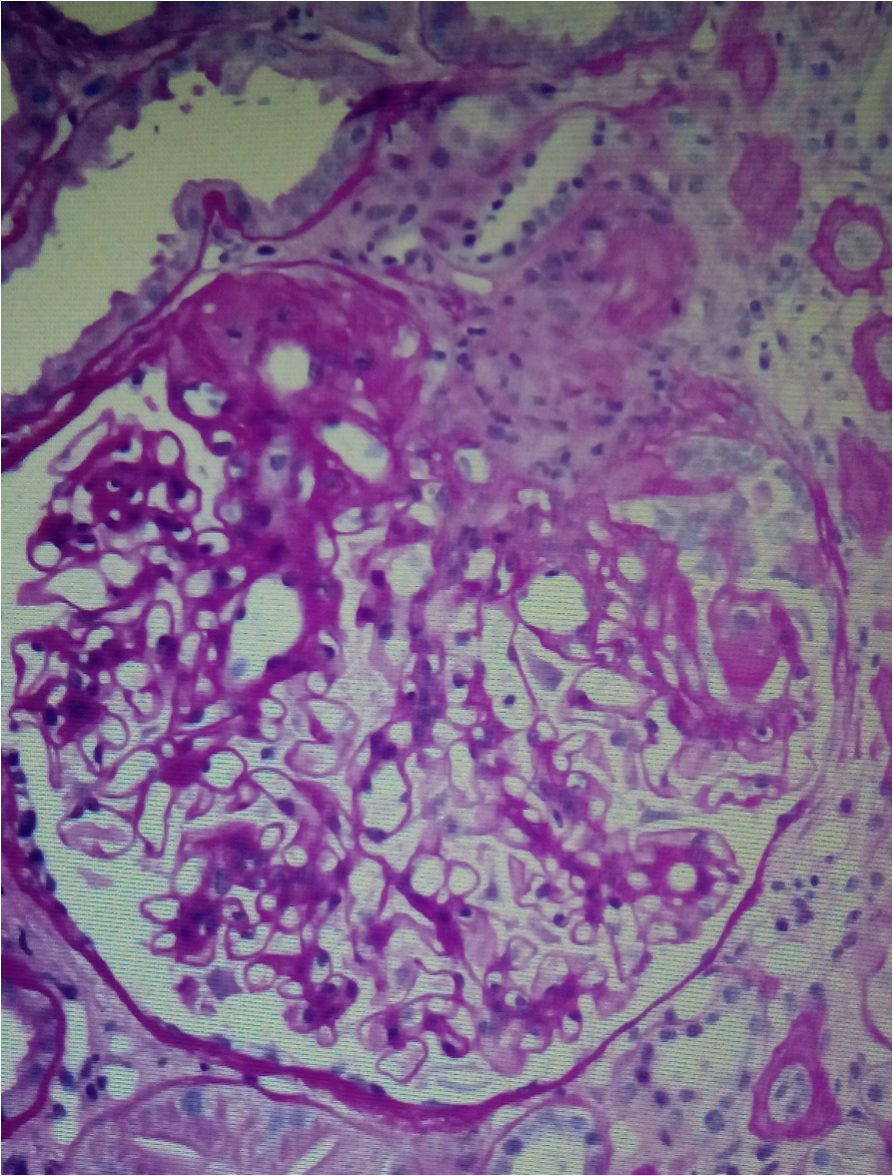
**High-power view of focal and segmental glomerulosclerosis (periodic acid–Schiff stain).**

In view of demonstrated urinary protein loss and presence of dyslipidaemia, a renal biopsy was performed. The renal biopsy (Figure 4) showed the presence of focal segmental glomerulosclerosis^a^ (FSGS); not otherwise specified. A total of 18 glomeruli were examined of which two showed segmental sclerosis with adhesion to Bowman’s capsule. Glomeruli were stained for immunoglobulin (Ig G, IgM, IgA, C3, C1q, kappa and lambda light chains. All were negative.

Thus the issue of anasarca was addressed and the patient was put on oral steroids. He responded very well and the steroids could be gradually tapered off without recurrence of symptoms. However, the issue of widespread lymphangiectasia remained unresolved even after thorough search and imaging analysis.

## Discussion

Chylous ascites refers to accumulation of milky peritoneal fluid in the abdominal cavity. High TG concentration is responsible for the nature of the fluid. TG levels are characteristically >200mg%, although usually the level turns out to be >1000mg% [1]. If the cholesterol content is high rather than TG, then the fluid is described as pseudochylous [2].

Lymphomas, metastatic malignancies, abdominal surgeries and infections like tuberculosis commonly cause chylous ascites [3]. The presence of chylous ascites has been rarely reported in nephrotic syndrome. Lindenbaum and Scheidt reported the existence of chylous ascites in nephrotic syndrome in 1968 [4]. The pathologic findings in their patients were membranous nephropathy, membranoproliferative nephropathy and FSGS. Chylous ascites in a background of lymphangiectasia usually signifies obstruction of flow through lymphatics. However, the existence of lymphangiectasia without any lymphatic obstruction in patients with chylous ascites has been reported in the literature as part of ‘yellow nail syndrome’ [5]. In our report, the patient was not found to have any nail abnormalities although lymphangiectasia could be demonstrated in his duodenum and spleen. A lymphangiogram did not reveal any lymphatic obstruction in this case; hence, the widespread lymphangiectasia was poorly explained. Chylous ascites could be explained by the fact that there are reports of adult onset nephrotic syndrome associated with chylous ascites; although the underlying exact pathogenesis remains elusive. Another curious aspect of this case is the presence of duodenal as well as splenic lymphangiectasia. The metabolic changes of nephrotic syndrome increase permeability of serosal and mucosal lymphatics which may have some connection with the lymphangiectasia described above. ‘Hennekam syndrome’, an uncommonly reported entity in literature, has the components of lymphedema and widespread lymphangiectasia [6]. However, this syndrome, which is thought to represent a developmental disorder of lymph vessels, presents with dysmorphic facial features and mental retardation. Hence, our case did not conform to the description of Hennekam syndrome. This is probably the first reported case to have chylous ascites, adult onset nephrotic syndrome with FSGS on renal histology and presence of lymphangiectasia in the same patient.

## Conclusions

Chylous ascites is usually caused by demonstrable obstruction in lymphatic outflow channels, resulting in extravasation of milky white fluid into the peritoneal cavity. Accumulation of chylous fluid in peritoneal space may occur, in rare instances, without any obvious demonstrable obstruction in lymph channels. Nephrotic syndrome is a rare cause of chylous ascites and has to be considered in the differential diagnoses while dealing with such clinical situations.

Ectasia of lymph vessels in a background of chylous ascites points towards an obstructive aetiology, although in rare situations, such as in this case, obstruction may not be demonstrable even after a thorough search, which makes the presented case a rarity. Thus, lymphangiectasia and chylous ascites may coexist without other demonstrable abnormality of lymph vessels.

## Consent

Written informed consent was obtained from the patient for publication of this case report and accompanying images. A copy of the written consent is available for review by the Editor-in-Chief of this journal.

## Endnotes

^a^On histological examination, FSGS is characterised by involvement of some, not all, of the glomeruli (focal). Some tufts within a particular glomerulus (segmental) show the lesion.

The affected glomeruli exhibit increased mesangial matrix, obliterated capillary lumens, and deposition of hyaline masses (hyalinosis) and lipid droplets. Occasionally, glomeruli are completely sclerosed (global sclerosis).

## Abbreviations

FSGS: Focal segmental glomerulosclerosis
GI: Gastrointestinal
IG: Immunoglobulin
TG: Triglyceride
